# Does the reading brain possess top-down connections from morphemes to letters?

**DOI:** 10.3758/s13423-026-03037-9

**Published:** 2026-10-27

**Authors:** Maša Mlinarič, Jonathan Grainger, Elisabeth Beyersmann, Joshua Snell

**Affiliations:** 1 Vrije Universiteit Amsterdam, Amsterdam, Netherlands; 2 Centre Nationale de Recherche Scientifique, Paris, France; 3 Macquarie University, Sydney, Australia

**Keywords:** Reading, Morphological processing, Orthographic processing

## Abstract

No psycholinguist will contradict the importance of morphemes in language processing. But as a cognitive construct, the morpheme and its operations are not fully mapped out yet. Here, we investigated whether morphemes provide top-down support for their constituent letters: a so-called morpheme superiority effect. In two experiments (*N* = 100 per experiment), French participants identified single letters in nonword strings that either comprised a French affix (e.g., ‘o’ in ‘cojuste’) or not (‘o’ in ‘sojuste’). In Experiment 1 participants provided free-recall responses by typing letters seen at postcued locations. Experiment 2 was a Reicher–Wheeler (i.e., two-alternative forced-choice) version of the same task. Neither experiment evidenced better identification of target letters embedded in affixes than nonaffixes. On the other hand, we did observe an impact of the frequencies of embedding N-grams (i.e., letter chunks). Hence, while the brain learns and represents letter combinations, we did not observe evidence for morpheme representations partaking in the brain’s feedback loop. Findings are discussed in light of the recent PONG model of word recognition.

**Supplementary Information:**

The online version contains supplementary material available at https://doi.org/10.3758/s13423-026-03037-9.

## Introduction

It is a fundamental principle of cognitive science that the brain achieves cognition not only via bottom-up but also top-down connections. This has been made particularly evident in the reading brain, with reports of so-called superiority effects at various levels of representation. For example, the word superiority effect (i.e., faster detection of ‘a’ in ‘man’ than in ‘nam’) evidences feedback from neurons coding for words to neurons coding for letters (e.g., Cattell, 1886; Reicher, 1969; Wheeler, 1970). Meanwhile, the sentence superiority effect (i.e., faster detection of ‘man’ in ‘the man can run’ than in ‘run man the can’) evidences sentence-to-word feedback (e.g., Cattell, 1886; Snell & Grainger, 2017; Wen et al., 2019; see also Fallon & Pylkkänen, 2024; Hossain & White, 2023; Snell, 2025a; Snell & Melo, 2024). Superiority effects have guided the development of a large variety of connectionist models, which capture phenomena through *interactive* *activation* among visual features and letters (McClelland & Rumelhart, 1981), words (e.g., Davis, 2010; Grainger & Van Heuven, 2003; Snell, 2025b; Whitney, 2001) and sentences (e.g., Lopes Rego et al., 2024; Seijdel et al., 2024; Snell et al., 2018).

**Fig. 1:**
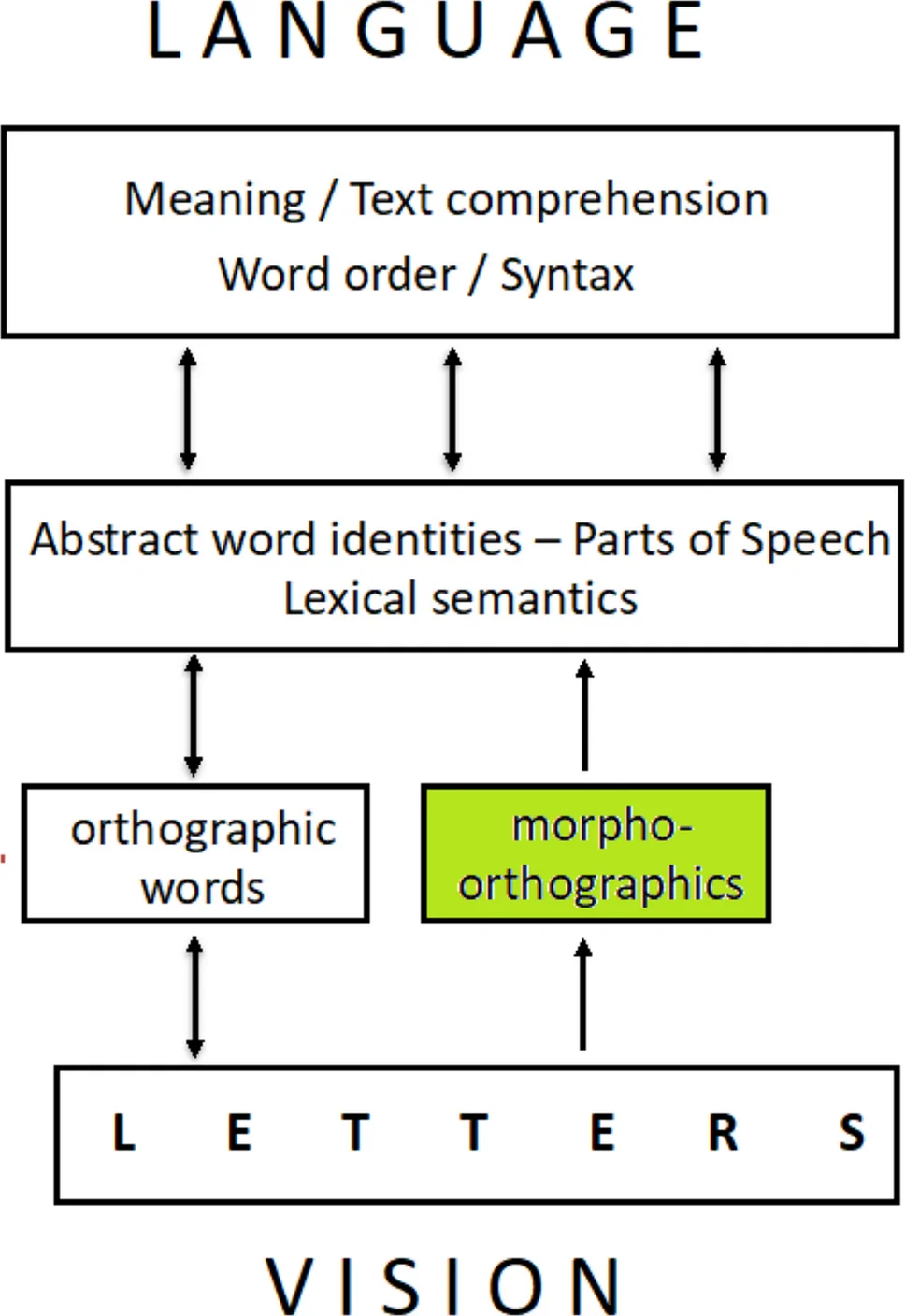
Model of the main processing stages involved in the transition from low-order vision to high-order comprehension, adapted from Grainger (2024). The morpheme representation is highlighted in green. Influences among all other linguistic units are bi-directional (reflected by the bi-directional arrows). The morpheme, in contrast, is activated by letters and in turn activates words, but neither receives top-down activation from words nor provides top-down activation to letters

Amidst letters, words and sentences, there is yet another linguistic unit that has hitherto been somewhat of an oddball: the morpheme. Defined as the smallest unit of meaning (e.g., Haspelmath & Sims, 2010), the morpheme is an oddball for various reasons. For one, it is not included in most models of word recognition (Davis, 2010; Gomez et al., 2008; Grainger & Jacobs, 1996; Grainger & Van Heuven, 2003; McClelland & Rumelhart, 1981; Snell, 2025a; Snell et al., 2018; Whitney, 2001). And, to date, the models that do include it are box-and-arrow illustrations without computational implementation (e.g., Beyersmann & Grainger, 2023; Taft, 1994; Grainger & Beyersmann, 2017; Grainger, 2024), thus precluding tests of theory through simulation. More importantly, the morpheme has been an oddball because, unlike other linguistic units, its conceptualizations do not invoke interactive activation—at least in a fully bi-directional sense. To illustrate, in the model of Grainger (2024; see Fig. 1), the morpheme is the only linguistic unit that is situated in a one-way street: the morpheme is activated by letters and in turn activates words, but neither receives top-down activation from words nor provides top-down activation to letters (see also Grainger & Beyersmann, 2017).^1^

An exception is the model of Taft (1994), which includes not only bottom-up connections from morphemes to words but also top-down connections from words to morphemes (see Fig. 2 in Taft, 1994). However, in the description of his model, Taft did not refer to top-down connections from morphemes to letters; only to bottom-up connections from letters to morphemes. The data discussed by Taft specifically spoke to the notion of interactions among morphemes and words, not interactions among morphemes and letters. And indeed, we reckon the general lack of ‘fully interactive’ theories of morphological processing is mainly because there is, as yet, a lack of empirical evidence to the contrary. More generally, while studies suggest that complex words are decomposed into constituent stem and affix representations, and that the ease of recognizing words depends on whether this decomposition succeeds (see “Morpheme Representations: Current Evidence” section), less is known—empirically speaking—about the affix’s subsequent operations. Additionally, as we will argue below, some effects that have been attributed to morphemes are difficult to disentangle from potential confounders, such as the statistical frequency distributions of letter combinations, which may directly impact word recognition (Snell, 2025a). In short, from an empirical point of view the morpheme remains somewhat mysterious. The present paper addresses this gap with a new angle on morphemes: By hypothesizing, for the first time, a morpheme superiority effect, we hope to reveal some of the morpheme’s operations in the brain, thereby giving the construct more substance.

**Fig. 2:**
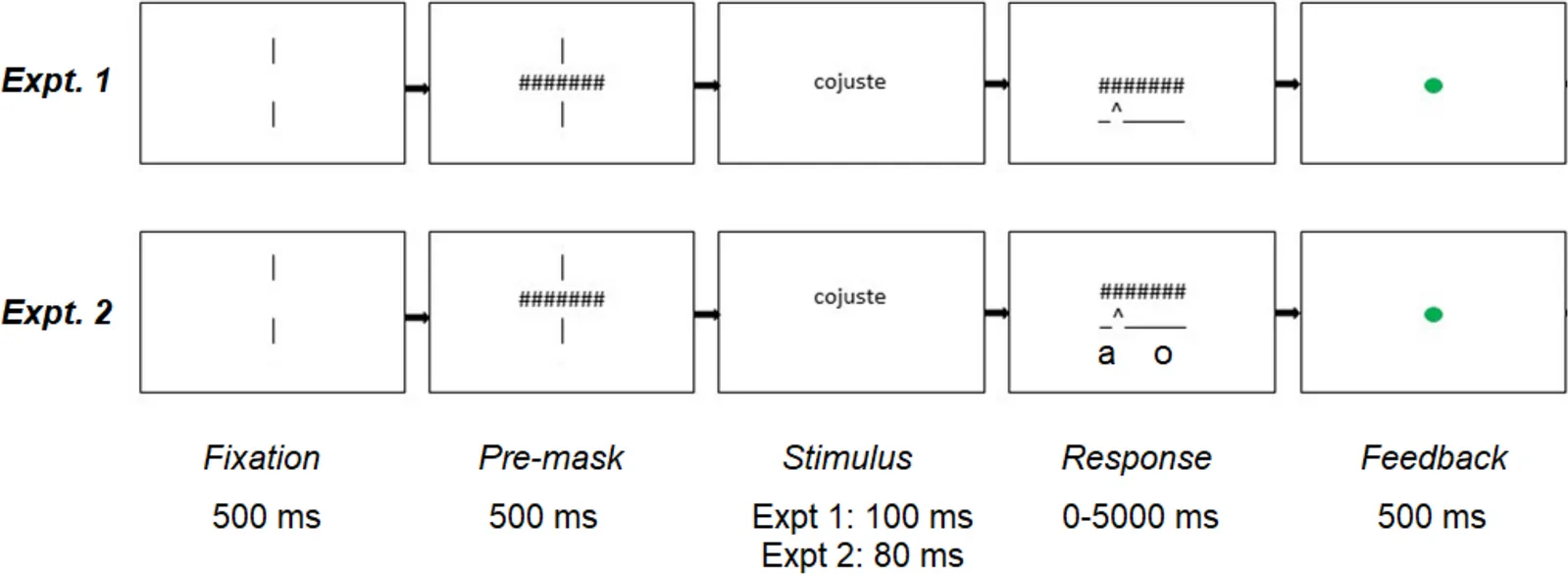
Schematic overview of a single trial in Experiment 1 (upper row) and 2 (bottom row)

### Morpheme representations: Current evidence

It is a longstanding notion that, upon viewing a complex word, the brain rapidly and automatically decomposes it into morpho-orthographic units (e.g., *‘farmer’* ➔ ‘*farm*’ + ‘*er*’). Priming studies constitute the primary empirical basis for this: lexical decisions about target words are made quicker when those targets are preceded by a brief (~50 ms) prime stimulus in which the target occurs as embedded stem alongside an affix (e.g., ‘*farmer*’ primes subsequent target ‘*farm*’)—and this effect is also observed with so-called ‘pseudo-suffixed’ primes, which contain letter chunks that might elsewhere serve as suffix but are not an actual suffix in the prime (e.g., ‘*wander*’, which contains suffix ‘*er*’, primes the semantically unrelated ‘*wand*’). That such priming effects are not simply caused by orthographic overlap is made clear by reduced (or absent) priming in the case of nonsuffixed words (e.g., ‘*cashew*’ does not prime ‘*cash*’; Amenta & Crepaldi, 2012; Beyersmann et al., 2016; Grainger et al., 2021; Longtin et al., 2003; Rastle et al., 2004).

Further evidence for early morpho-orthographic segmentation comes from electro-encephalography. The typical finding is that event-related potentials (ERPs) caused by truly suffixed and pseudo-suffixed words differ from those caused by nonsuffixed words in early time windows (~150–200 ms), while the ERPs for truly suffixed and pseudo-suffixed words themselves only differ in later time windows (~250–400 ms; e.g., Beyersmann et al., 2019; Lavric et al., 2007, 2011, 2012; Morris et al., 2008).^2^

Thus, whether informed by behavioural or neurophysiological data, all conclusions somehow hinge on contrasts among truly affixed, pseudo-affixed and nonaffixed words. And herein lies a problem. Across all studies to date, words from these three categories vary in their frequency (perhaps inevitably so). In the studies of Lavric et al. (2007, 2011), truly affixed, pseudo-affixed and nonaffixed target frequencies were, respectively, ~48, ~42 and ~36 parts per million,^3^ which may be considered reasonably equal. But differences are more pronounced when considering not only entire words but also letter clusters within words. For example, between the suffixed ‘*cashing*’ and nonsuffixed ‘*cashew*’, the N-gram ‘*ing*’ is nearly ten times as frequent as the N-gram ‘*ew*’, at ~2,081 and ~225 ppm, respectively. N-gram frequencies have not been reported in any of the aforementioned studies; but according to recent theories of word recognition, these N-gram frequencies heavily impact lexical activation (Snell, 2025b). In the present study we mitigate this issue by including N-gram frequencies as factors in our analysis models.

If we assume, in spite of aforementioned confounder, that the affix is indeed ‘stripped off’ (i.e., that complex words activate dedicated affix representations), what would that affix do subsequently? Common belief is that the brain affords connections among truly affixed lexical representations (e.g., ‘*farmer*’), semantic representations (e.g., all farm-related things) and a semantic representation of the affix (‘*er*’), whereby the affix provides feedback from the semantic to the lexical level (e.g., Beyersmann & Grainger, 2023). Evidence for this consists of slightly easier recognition of words that are preceded by truly suffixed primes than pseudo-suffixed primes, specifically when those primes are presented for a longer amount of time (Lavric et al., 2011). The idea here is that it takes some time for semantic feedback to kick in; hence, the contingency on prime duration (see also Beyersmann et al., 2020). Further, in their seminal study, Marslen-Wilson et al. (1994) reported evidence for suffix-to-suffix interference (e.g., ‘*government*’ primes ‘*govern*’ but not ‘*governor*’).

While the experiments of Marslen-Wilson et al. (1994) showed *lateral* connectivity, there is little evidence to suggest *top-down* connectivity whereby affix representations impact the ongoing recognition of their constituent letters. There are however a few studies that tentatively speak to this specific issue. Beyersmann et al. (2015) and Hasenäcker et al. (2021) conducted letter search tasks where target letters were embedded either in an affix (in an otherwise nonlexical stimulus string, e.g., *harbing*) or in a nonaffix (e.g., *harbong*). Beyersmann et al. (2015) observed worse detection of target letters embedded in an affix, but specifically when it concerned a suffix; no effects were found for prefixes. The detrimental effect was interpreted as a suffix-chunking effect, whereby identification of the suffix as a higher-level linguistic unit would trigger inhibition of its constituent letters so as to expedite processing of the stem. Notably, while the suppression of the constituent letters of affixes would have functional merit by this rationale, it is also precisely the opposite of what one would predict under the general notion of interactive activation (by which higher-level units should *activate* rather than *inhibit* constituents). Conversely, Hasenäcker et al. (2021) found some evidence for a morpheme superiority effect (i.e., better detection of target letters embedded in affixes) in one of their experiments, but only in an adult sample (meanwhile, no effects were found in samples of children from Grades 3 and 5); and critically, this effect was not replicated in a follow-up experiment. As argued by Hasenäcker et al. (2021) themselves, a letter search task may not be the perfect setting for investigating letter-in-morpheme processing, because this task may engender processing strategies that are quite unlike word reading (prompting, for instance, serial search instead of more holistic processing).

In the present study we instead employ a postcued letter-in-string identification task, analogous to the Reicher–Wheeler setting through which the classic word superiority effect is traditionally revealed (Reicher, 1969; Wheeler, 1970). The key difference from the letter search tasks that were employed by Beyersmann et al. (2015) and Hasenäcker et al. (2021) is that our task should promote processing of all letters (given that participants do not know which of the letters will be probed). By contrast, in the letter search task, the brain may be mostly sensitive to target letter features while suppressing and/or ignoring nontarget letters. We reckon this would likely hinder chances of observing a morpheme superiority effect, where those nontarget letters play a key role.

### The present study

If affix representations exist, and if these—akin to other linguistic units—obey interactive activation principles, then we may predict a morpheme superiority effect, whereby letter recognition is aided by morpheme activation. Here we put this hypothesis to the test in two experiments: one employing a free recall task, and the other employing a two-alternative forced-choice task in the spirit of Reicher–Wheeler. Because the experiments were overlapping in most respects and Experiment 2 mostly served as a replication, we present them jointly—with a focus on Experiment 1. All data and analysis scripts are openly available at https://osf.io/8z43h/.

## Methods

### Participants

The Prolific platform (www.prolific.com) was used to recruit 100 participants for each experiment (mean age ≈ 32 and 33 years in Experiments 1 and 2, respectively). All participants were registered as adult nondyslexic native French readers with normal or corrected-to-normal vision, and a Prolific ‘approval rate’ above 94%. Participants provided their informed consent prior to participating in either of the experiments and were compensated £3.50 for their time. The experiments were approved by the Ethics committee of the Faculty of Behavioral and Movement Sciences of the Vrije Universiteit Amsterdam.

### Stimuli

We used the stimulus set from the study of Beyersmann et al. (2015), which comprised 72 nonword strings with an affix (36 prefixes, 36 suffixes), paired with 72 nonaffixed nonwords that differed from their affixed counterpart by one letter (e.g., *cojuste* vs. *sojuste*). Stimuli were six to eight letters long (*M* = 6.92). The stimulus set also comprised 72 nonword filler stimuli, making for a total of 214 trials. All stimuli are provided in the Appendix.

For each affixed nonword and its nonaffixed counterpart we calculated the average frequency of constituent letter clusters that comprised the target letter (specifically, 2-grams, 3-grams, 4-grams, and 5-grams; the frequencies of longer N-grams were considered negligible) by counting all occurrences of each N-gram in the French component of the *subs2vec* database of van Paridon and Thompson (2021), and transforming these counts to log ppm. N-gram frequencies were added as covariate in the analyses. It is worth noting that there was in fact an unavoidable but considerable difference in N-gram frequency between affixed and nonaffixed strings: the former were on average 1.10 log ppm more frequent than the latter. Had we not taken N-gram frequencies into account, this would have constituted a confound.

In Experiment 2, upon probing the target letter position, we presented the target letter alongside an arbitrarily chosen but orthographically legal foil letter.

### Design

We tested participants’ letter detection speed in two experimental conditions: letters were embedded either in an affix (e.g., the ‘o’ in *cojuste*) or not (e.g., the ‘o’ in *sojuste*). In prefixed strings and their nonaffixed counterparts, we always probed recognition of the second letter. In suffixed strings and their nonaffixed counterparts, we always probed recognition of the penultimate letter (e.g., the ‘u’ in *mieleur* vs. *mieleuf*). In the filler stimuli we probed letters at intervening positions (e.g., the ‘u’ in *élfubie*), so that participants would not develop attentional biases to the edges of the stimulus. The total of 214 trials was presented in random order.

### Procedure

The experiments were implemented with OpenSesame (Mathôt et al., 2012) and run online on the JATOS servers (Lange et al., 2015). Participants were instructed to do the experiment on their personal laptop.

The experiments took approximately 20 min. Each trial started with a 500-ms fixation screen followed by a 500-ms premask composed of hashtags (see Fig. 2). Then a nonword string was presented for 100 ms in Experiment 1 and 80 ms in Experiment 2 (because we deemed the latter experiment to be easier), and followed by a postmask, which stayed onscreen until the participant’s response. Below the postmask, an upward arrow (constituted by the “^” sign) indicated which letter needed to be reported. Masks and nonword strings were presented in 22px monospaced font. In both experiments participants had a maximum of 5,000 ms to press a letter key to indicate which letter they had seen at the corresponding location. In Experiment 2, the target and foil letter were presented 128 px below the mask, on the left and right side of the display at a 128 px distance from one another, and participants had to press the arrow left or right key to choose the side of the target letter. The positions of target and foil were randomized on each trial. If no response was given, the trial was marked as incorrect. Feedback with a 500-ms green or red dot was provided at the end of the trial for correct or incorrect answers, respectively. The 214 experimental trials were preceded by 10 practice trials.

## Results

For Experiment 1, from the initial 100 participants we replaced 21 that had an overall accuracy below 60%, and one other participant that had accidentally done the experiment twice. From Experiment 2 we replaced 11 participants who had accuracies below 60% and seven who had not provided responses during the experiment. We found out that one of the prefixed stimuli was accidentally nine letters long, whereas all other stimuli were six to eight letters long; so, we excluded the nine-letter stimulus from the analyses. We thus ended up with 7,100 measurements per experimental condition in each experiment. Q–Q plots revealed that the data were not normally distributed. We therefore log-transformed RTs for our analyses (but plot nontransformed RTs in the graphs below). We first excluded physiologically impossible trials (RT < 100 ms) and trials without a response. Subsequently, we excluded trials with a log-RT beyond 2.5 standard deviations from the individual participant’s mean log RT, whereby the mean and standard deviation were calculated exclusively on the basis of correctly answered nonfiller trials. For the analyses of RTs we also removed incorrectly answered trials. In Experiment 1, we excluded a total of ~13% of nonfiller trials from the RT analyses, and ~3% from the accuracy analyses. In Experiment 2, ~18% of trials were excluded from the RT analyses, and ~3% from the accuracy analyses. Filler trials were not analysed.

Linear mixed-effects models (LMMs) and generalized LMMs (gLMMs) were used to analyse RT and accuracy, respectively. Models were fitted with the lmer() and glmer() functions of the *lme4* package (Bates et al., 2015) in *R* (R Core Team, 2023). Models included morpheme presence (*present* vs. *absent*) and N-gram frequency (continuous) as fixed effects, and item labels and participant as clustering variables. Affixed strings and their nonaffixed counterparts had the same item label. We always started with by-participant and by-item random intercepts and slopes for the morpheme presence condition, but simplified models in case there were convergence or singularity issues.^4^ For our main analyses of RTs this resulted in models without the by-subject random slope. Full random effects models were used for the accuracy analyses. Evidence for the null was quantified with Bayes Factors (BF) via $B{F}_{01}\approx {\sqrt{n}*e}^{- \frac{x^2}{2}}$, with *n* referring to the number of observations and *x* referring to the *t* or *z* value outputted by LMMs and gLMMs respectively, and assuming a unit-information prior (Wagenmakers, 2007).

We report *b* values, *SE*s and *t* (LMMs) or *z* values (gLMMs). Condition differences were interpreted as significant when | *t* | or | *z* | > 1.96. Nonaffixed trials were taken as a reference.

### Experiment 1

**Fig. 3:**
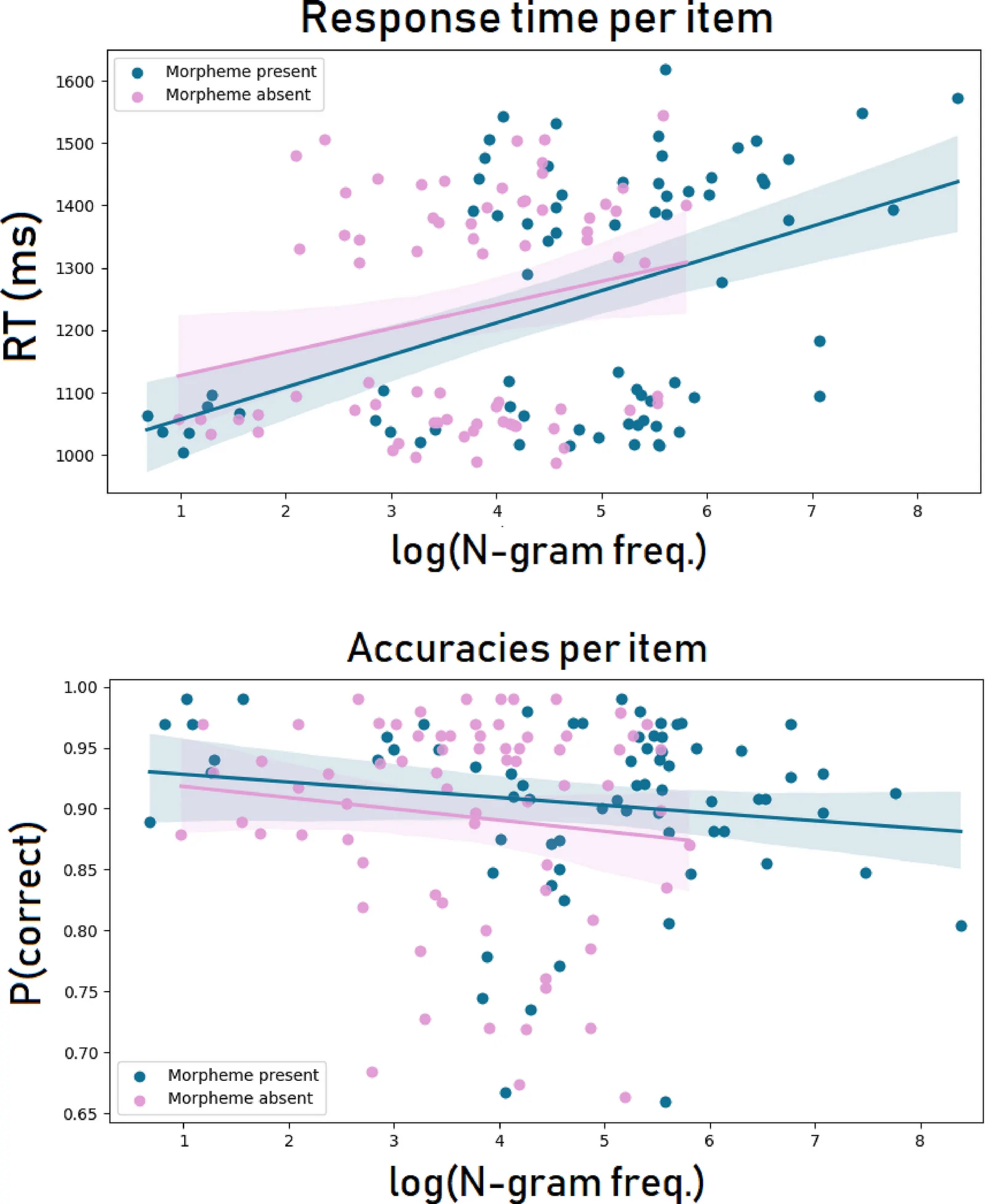
Average response times and accuracies for items with (blue) versus without (pink) morphemes for Experiment 1, whereby we also indicate the N-gram frequency of each item. Trend lines indicate effects of N-gram frequency on RT and accuracy, plotted separately for morpheme present (blue) versus morpheme absent (pink) trials. Shaded areas around the trend lines reflect 95% CIs. (Colour figure online)

Average RTs and accuracies for trials with and without morphemes in Experiment 1 are presented in Fig. 3. Contrary to our hypothesis, we observed no effects of morpheme presence, RT: *b* = 0.007, SE = 0.005, *t* = 1.27 (BF_01_ = 49.74); accuracy: *b* = 0.10, SE = 0.11, *z* = 0.88 (BF_01_ = 79.75). On the other hand, in both RT and accuracy we observed effects of N-gram frequency, with higher embedding N-gram frequency generally yielding lower performance (RT: *b* = 0.008, SE = 0.003, *t* = 2.69; accuracy: *b* = −0.09, SE = 0.05, *z* = −1.99).^5^

One might note in the upper panel of Fig. 3 fact that morpheme presence correlates considerably with N-gram frequency. Indeed, as noted in the “Stimuli” section, the N-gram frequency of affixed strings was ~1.10 log ppm higher than that of nonaffixed strings (*r*_*pb*_ = 0.36; *p* <.001); and all strings that had frequencies >6 log ppm exclusively contained an affix. We therefore reanalysed a subset of the data for which the average N-gram frequency between the morpheme *present* vs. *absent* was equal. In order to do this, we ordered our 71 items on the basis of each item’s frequency difference (morpheme *present* minus morpheme *absent*), and then excluded, in one-by-one fashion, as many items as needed to make the average frequency equal between conditions. Equality was reached after excluding 33 item labels, meaning this post hoc analysis was based on ~54% of trials for ~3,800 measurements per condition. Outlier exclusion procedures were run afresh with this new dataset prior to analysis. We again observed no effect of morpheme presence (RT: *b* = 0.007, SE = 0.005, *t* = 1.28, BF_01_ = 36.1; accuracy: *b* = −0.004, *SE* = 0.125, *z* = −0.03, BF_01_ = 85.80).

### Experiment 2

In Experiment 2, we largely replicated the patterns observed in Experiment 1. We again observed no effect of morpheme presence in RT (*b* = 0.003, *SE* = 0.007, *t* = 0.47; BF_01_ = 97.0) and accuracy (*b* = 0.079, *SE* = 0.073, *z* = 1.09; BF_01_ = 64.7). In the full model, with both morpheme presence and N-gram frequency included, N-gram frequency did not have a significant effect in RT (*b* = 0.005, *SE* = 0.004, *t* = 1.48) or accuracy (*b* = −0.005, *SE* = 0.034, *z* = −0.15). However, as elaborated in “Experiment 1” section, our two fixed effects were highly collinear, which, we reckoned, precluded proper testing of the influence of N-gram frequency. We therefore simply assessed this effect separately in the morpheme-present and morpheme-absent trials, so that no variance caused by N-gram frequency could be erroneously attributed to morpheme presence. Significant effects of N-gram frequency were observed in the morpheme-present trials (RT: *b* = 0.023, *SE* = 0.007, *t* = 3.26; accuracy: *b* = −0.145, *SE* = 0.061, *z* = −2.37) but not the morpheme-absent trials (RT: *b* = 0.014, *SE* = 0.011, *t* = 1.26; accuracy: *b* = 0.020, *SE* = 0.084, *z* = 0.23). The effects observed in morpheme-present trials were beyond a Bonferroni-corrected significance threshold of | *t* | and | *z* | ≥ 2.24 (we used this increased significant threshold since we essentially tested the hypothesis twice). We reckon that the N-gram frequency effect was exclusively observed in morpheme-present trials because frequency values were less dispersed (and systematically lower) in the morpheme-absent trials (in the cleaned data, for Morpheme present trials, N-gram frequency in log(ppm) was M = 4.7, SD = 1.7; for morpheme-absent trials, meanwhile, *M* = 3.7, *SD* = 1.1).

Similarly to the post hoc analysis of Experiment 1, we also tested effects of morpheme presence with the subset of stimuli for which N-gram frequencies were equal between trials with and without affix. Again, no effects were observed (RT: *b* = 0.007, *SE* = 0.007, *t* = 0.94, BF_01_ = 51.0; accuracy: *b* = 0.103, *SE* = 0.071, *z* = 1.44, BF_01_ = 30.3).

## Discussion

Here we explored the existence of a morpheme superiority effect, whereby activated morpheme representations would provide top-down support of their constituent letter representations; analogous to the well-known word superiority effect. In the absence of prior empirical tests of this scenario, morphemes have long been the ‘odd one out’ in theories of the reading brain: most contemporary models show bi-directional connections among letters, words, and sentences (e.g., Grainger, 2024; Snell et al., 2018), but the morpheme is positioned in the middle of a one-way-street from letters to words (Grainger, 2024; Fig. 1; but see Taft, 1994, in which top-down connections from morphemes to letters could be possible but are not explicitly assumed).

Judging by the present results, the assumption of a one-way-street may well have been correct: Target letters were not recognized better when embedded in a morpheme.^6^ Meanwhile, we did observe an influence from the frequencies of embedding N-grams, with higher embedding N-gram frequency leading to slower and less accurate target recognition (e.g., detection of the ‘O’ is better in ‘QOZU’ than in ‘LORA’). This particular finding is informative both from a methodological and a theoretical point of view. Methodologically, it suggests that our design afforded enough sensitivity to be able to detect influences from surrounding letters on target letter recognition (but it should be noted that in Experiment 2 we only found frequency effects in the morpheme-present trials, in which the frequency variable indeed varied more). Theoretically, the impact of embedding N-gram frequency—while not our main result of interest—does provide support for one recent model of word recognition, PONG (Snell, 2025b; Snell & Simon, 2025). According to PONG (which describes the *P*ositional *O*rdering of *N-G*rams), the brain represents not only single letters, but also letter combinations of various sizes, depending on how frequently those combinations are encountered in the environment. Importantly, there is no hierarchy among N-grams of different sizes (e.g., 3-grams are neither activated by, nor reciprocally activate, 2-grams or 1-grams). Instead, all these N-grams jointly constitute the layer of sublexical orthographic processing, and connect to embedding word representations. Taking the above examples ‘QOZU’ and ‘LORA’, in the brain of an English reader ‘QOZU’ would lead to strong activation of ‘O’ and ‘U’ because these are frequent N-grams; but ‘QO’, ‘QOZ’, ‘OZ’, ‘OZU’ and ‘QOZU’ are hardly activated, if at all. Meanwhile, ‘LORA’ triggers more activation of a larger number of N-grams, because the brain has, through numerous encounters, developed a representation for them. The benefit that we observed for ‘QOZU’ would in this case stem from the fact that the target, ‘O’, is one of but a few strongly activated N-grams, and can therefore be read out more easily. Importantly, this also aligns with the study of Beyersmann et al. (2015), who found better detection of target letters embedded in low-frequency affixes.

Interestingly, PONG does not assign a special role to morphemes. For all intents and purposes, within PONG morphemes are just relatively frequent N-grams. Given that the present experiment revealed an effect of N-grams but not of morphemes, could it be that the brain simply does not possess dedicated morpheme representations at all? Unfortunately, the present data do not provide a conclusive answer: There is still the alternative possibility that morphemes do exist in the brain, but that they do not provide feedback activation of letters, as assumed in the model of Grainger (2024). That being said, as argued in the Introduction, the fact remains that much of the prior evidence for morpheme representations can be explained through N-gram frequencies (see, e.g., Chetail, 2017; Lelonkiewicz et al., 2020, 2023, for evidence that, at least at a certain level of analysis, morphemes are simply frequent chunks of letters). Had we observed a morpheme superiority effect, this would have helped to substantiate the morpheme as a cognitive construct; but this goal was not borne out by the data.

On a final theoretical note, how likely is it that the brain has dedicated morpheme representations in addition to letter and word representations, but that top-down connections exclusively exist between words to letters (known since the seminal studies of Reicher, 1969, and Wheeler, 1970) while being absent between morphemes and letters? One could potentially argue that the former has functional merit whereas the latter would not. Considering the classic word superiority effect, for example, upon seeing the word ‘WORK’ suboptimally, with uncertainty about the final letter (‘WORK’ vs. ‘WORD’), activation of the corresponding lexical representation would aid ongoing recognition of the ‘K’. This functional significance is perhaps not so obvious in the case of morpheme-to-letter feedback. Consequently, one could conceive of an interactive-activation framework that selectively affords top-down connections only where such connections have functional merit. Ours is a different theoretical stance, however. Our study is motivated by the fact that the notion of interactive activation—and recurrent feedback in specific—could in principle be applied as a general property of the brain’s architecture. In a vast amount of ways recurrent feedback would have functional merit; and then in a few other ways it may have little value or even be detrimental (e.g., when a priori predictions lead to erroneous recognition of unexpected things). On the whole, then, the net value of recurrent feedback *as a general principle* is still positive; and if it is indeed a general principle of the brain’s architecture that representations feedback to constituents, we may expect to find it also in places where there is no direct apparent benefit. Naturally, the human brain did not evolve with the specific purpose of representing morphemes that feed back to letters; but if today’s brains represent morphemes, and if interactive activation as a general principle holds, then we may well have expected morpheme-to-letter feedback (for better or worse).

In conclusion, we observed no evidence for feedback activation from morphemes to letters, which would be in line with a scenario wherein morpheme representations do not exist, or with a scenario wherein their connection to constituent letters is unidirectional, and exclusively feedforward, from letters to morphemes.

## Supplementary Information

Below is the link to the electronic supplementary material.

Supplementary file1 (DOCX 27 KB)

## Data Availability

All data and scripts are openly available online (https://osf.io/8z43h/).

## Footnotes

1 Note, some models depict supra-lexical ‘morpho-semantics’ rather than sublexical ‘morpho-orthographics’ (e.g., Diependaele et al., 2009). For the purpose of the present study these are quite similar: in neither case morphemes are assumed to provide feedback activation to the letter level.

2 Note, patterns may vary depending on whether primes are used, and if so, for how long those primes are presented onscreen (see, e.g., Leminen et al., 2019, for a review).

3 These authors reported a lack of significant frequency differences among the three conditions with a standard ANOVA. However, while the truly affixed and pseudoaffixed words’ frequencies were quite similar, the nonaffixed words’ frequency was ~25% lower.

4 In these cases, the slope that was highly correlated with the intercept was omitted first. In case both slopes were highly correlated with their corresponding intercepts, we omitted the one that contributed the least amount of variance.

5 Prompted by a reviewer, we ran a separate model to inspect the *N*-gram frequency effect per N-gram size (i.e., including bigram, trigram, quadrigram, and pentagram frequencies as separate IVs), and found that, in RTs, the effect was specifically driven by the trigrams (*b* = 0.009, *SE* = 0.003, *t* = 2.52). The frequencies of differently sized N-grams had no significant impact on RT (all values | *t* |< 0.46). Analysing accuracy in a same fashion, this model suggested that the detrimental frequency effect was driven by trigrams (*b* = −0.13, *SE* = 0.05, *z* = -2.53) as well as bigrams (*b* = −0.32. *SE* = 0.13, *z* = −2.41), but also yielded a smaller opposite effect of pentagrams (*b* = 0.06, *SE* = 0.02, *z* = 2.35).

6 Here it may also seem appropriate to acknowledge that evidence of absence principally does not exist (i.e., we have not directly evidenced the nonexistence of top-down morpheme-to-letter connections).

## Code availability

All data and scripts are openly available online (https://osf.io/8z43h/).
