# Supplementary material for: Does the reading brain possess top-down connections from morphemes to letters?
Source: Psychon Bull Rev. 2026 Oct 27;33(8):268. doi: 10.3758/s13423-026-03037-9 (PMC13714072; doi:10.3758/s13423-026-03037-9)
Supplement: Supplementary file 1 [file 13423_2026_3037_MOESM1_ESM.docx]

**Appendix**

*Stimuli*

*N.B.: These are the experimental stimuli. Filler stimuli are presented at the bottom of this Appendix.*

| ***With morpheme*** | ***Without morpheme*** | ***Target letter*** | ***Target location*** | ***Foil letter in Expt. 2*** |
| --- | --- | --- | --- | --- |
|  |  |  |  |  |
| cojuste | sojuste | O | Second | A |
| cobleu | sobleu | O | Second | A |
| copièce | sopièce | O | Second | A |
| cosale | sosale | O | Second | U |
| cograve | sograve | O | Second | U |
| copierre | sopierre | O | Second | U |
| renoir | penoir | E | Second | O |
| regros | pegros | E | Second | O |
| revrai | pevrai | E | Second | O |
| resoir | pesoir | E | Second | U |
| remain | pemain | E | Second | U |
| repaix | pepaix | E | Second | U |
| propoche | cropoche | R | Second | L |
| prodoux | crodoux | R | Second | L |
| propoint | cropoint | R | Second | L |
| procause | crocause | R | Second | I |
| proface | croface | R | Second | I |
| projambe | crojambe | R | Second | I |
| imroute | emroute | M | Second | G |
| imfois | emfois | M | Second | R |
| imoeil | emoeil | M | Second | R |
| imchien | emchien | M | Second | R |
| imrobe | emrobe | M | Second | L |
| imville | emville | M | Second | R |
| préfille | créfille | R | Second | N |
| prépaille | crépaille | R | Second | N |
| prébeau | crébeau | R | Second | N |
| préboîte | créboîte | R | Second | L |
| préchose | créchose | R | Second | L |
| prédame | crédame | R | Second | L |
| distour | vistour | I | Second | E |
| distête | vistête | I | Second | E |
| discher | vischer | I | Second | E |
| dismère | vismère | I | Second | A |
| disgosse | visgosse | I | Second | A |
| discarte | viscarte | I | Second | A |
| mieleur | mieleuf | U | Penultimate | I |
| verteur | verteuf | U | Penultimate | I |
| boiseur | boiseuf | U | Penultimate | I |
| chateur | chateuf | U | Penultimate | I |
| pleineur | pleineuf | U | Penultimate | I |
| parteur | parteuf | U | Penultimate | I |
| brasion | braseon | O | Penultimate | E |
| cielion | cieleon | O | Penultimate | E |
| haution | hauteon | O | Penultimate | E |
| piedion | piedeon | O | Penultimate | U |
| prêtion | prêteon | O | Penultimate | U |
| fleurion | fleureon | O | Penultimate | U |
| couprie | coupgie | I | Penultimate | O |
| ballerie | ballegie | I | Penultimate | O |
| jeunerie | jeunegie | I | Penultimate | O |
| forcerie | forcegie | I | Penultimate | C |
| fauterie | fautegie | I | Penultimate | C |
| placerie | placegie | I | Penultimate | C |
| sangure | sangire | R | Penultimate | G |
| filmure | filmire | R | Penultimate | G |
| goûture | goûtire | R | Penultimate | G |
| chefure | chefire | R | Penultimate | S |
| faimure | faimire | R | Penultimate | S |
| loupure | loupire | R | Penultimate | S |
| coinade | coinide | D | Penultimate | R |
| jourade | jouride | D | Penultimate | N |
| trainade | trainide | D | Penultimate | R |
| bruitade | bruitide | D | Penultimate | R |
| courade | couride | D | Penultimate | R |
| mortade | mortide | D | Penultimate | R |
| toutoir | toutois | I | Penultimate | U |
| fortoir | fortois | I | Penultimate | U |
| peuroir | peurois | I | Penultimate | U |
| bordoir | bordois | I | Penultimate | U |
| planoir | planois | I | Penultimate | A |
| longoir | longois | I | Penultimate | A |

*2. Filler stimuli*

| ***Filler stimulus*** | ***Target letter*** | ***Target position*** | ***Foil letter in Expt. 2*** |
| --- | --- | --- | --- |
|  |  |  |  |
| ebcume | C | 3 | R |
| pinume | N | 3 | A |
| lucume | C | 3 | A |
| osmugé | M | 3 | R |
| fomite | M | 3 | U |
| jamise | M | 3 | U |
| aneral | E | 3 | O |
| gamest | E | 4 | O |
| tafelt | E | 4 | O |
| daines | N | 4 | R |
| zaisen | S | 4 | R |
| kairen | R | 4 | T |
| rapsune | S | 4 | L |
| farlune | L | 4 | T |
| énudate | D | 4 | T |
| londume | D | 4 | T |
| bloine | I | 4 | U |
| élfunie | U | 4 | A |
| plipene | P | 4 | C |
| vatoine | T | 3 | R |
| fâchuné | C | 3 | R |
| menurbe | N | 3 | T |
| flacine | C | 4 | M |
| bantues | N | 3 | L |
| édugai | G | 4 | N |
| gudare | D | 3 | N |
| psudée | D | 4 | N |
| adalisé | A | 3 | E |
| padicral | D | 3 | N |
| odinage | I | 3 | U |
| peugré | U | 3 | N |
| émucré | U | 3 | I |
| éfroumé | R | 3 | L |
| cevoibe | O | 4 | E |
| ablouse | O | 4 | E |
| gribove | B | 4 | M |
| ebcure | C | 3 | I |
| pinule | N | 3 | C |
| lucuse | C | 3 | G |
| ostugé | T | 3 | R |
| folite | L | 3 | R |
| jagise | G | 3 | P |
| anural | U | 3 | E |
| gamast | A | 4 | E |
| tafilt | I | 4 | O |
| dainus | N | 4 | R |
| zaisan | S | 4 | R |
| kairun | R | 4 | S |
| rapsure | S | 4 | L |
| farlude | L | 4 | T |
| éludate | D | 4 | N |
| lordume | D | 4 | S |
| bloipe | I | 4 | U |
| élfubie | U | 4 | O |
| plipere | P | 4 | N |
| vatoile | T | 3 | G |
| fâchuté | C | 3 | R |
| meturbe | T | 3 | B |
| flacibe | C | 4 | R |
| bartues | R | 3 | L |
| énugai | G | 4 | M |
| gusare | S | 3 | N |
| psumée | M | 4 | L |
| apalisé | A | 3 | E |
| pasicral | S | 3 | V |
| ominage | I | 3 | E |
| peuglé | U | 3 | I |
| émuclé | U | 3 | I |
| éfloumé | L | 3 | R |
| ceveibe | E | 4 | U |
| abliuse | I | 4 | E |
| gribive | B | 4 | C |
